# Cumulative smoking exposure, duration of smoking cessation, and peripheral arterial disease in middle-aged and older Korean men

**DOI:** 10.1186/1471-2458-11-94

**Published:** 2011-02-11

**Authors:** Young-Hoon Lee, Min-Ho Shin, Sun-Seog Kweon, Jin-Su Choi, Jung-Ae Rhee, Hye-Ran Ahn, Woo-Jun Yun, So-Yeon Ryu, Bok-Hee Kim, Hae-Sung Nam, Seul-Ki Jeong, Kyeong-Soo Park

**Affiliations:** 1 Regional Cardiocerebrovascular Center, Chonnam National University Hospital, Gwangju 501-757, Republic of Korea; 2 Department of Preventive Medicine, Chonnam National University Medical School, Gwangju 501-746, Republic of Korea; 3 Jeonnam Regional Cancer Center, Chonnam National University Hwasun Hospital, Jeollanamdo 519-809, Republic of Korea; 4 Department of Preventive Medicine, Chosun University College of Medicine, Gwangju 501-759, Republic of Korea; 5 Department of Food and Nutrition, College of Natural Sciences, Chosun University, Gwangju 501-759, Republic of Korea; 6 Department of Preventive Medicine, Chungnam National University College of Medicine, Daejeon 301-747, Republic of Korea; 7 Department of Neurology, Chonbuk National University Medical School, Jeonju 561-180, Republic of Korea; 8 Department of Preventive Medicine, Seonam University College of Medicine, Jeollabukdo 590-711, Republic of Korea

## Abstract

**Background:**

We investigated the association of cumulative smoking exposure and duration of smoking cessation with peripheral arterial disease (PAD).

**Methods:**

The study population consisted of 2517 community-dwelling Korean men aged 50 years and older. Information on smoking characteristics such as smoking status, pack-years of smoking, and years since quitting smoking was collected using a standardized questionnaire. PAD was defined as an ankle-brachial index (ABI) less than 0.90 in either leg.

**Results:**

The odds ratio (OR, 95% confidence interval) of PAD was 2.31 (1.20-4.42) for former smokers and 4.30 (2.13-8.66) for current smokers, after adjusting for other cardiovascular risk factors. There was a significant dose-response relationship between pack-years of smoking and PAD. Compared with those who had never smoked, the multivariate-adjusted ORs of PAD for smokers of 0.1-20.0, 20.1-40.0, and >40.0 pack-years were 2.15 (1.06-4.38), 2.24 (1.08-4.65), and 2.93 (1.41-6.09), respectively. There was a significant decrease in PAD risk as the years since quitting smoking increased. The multivariate-adjusted ORs of PAD for 11-20 and ≥21 years smoking cessation were 0.41 (0.19-0.86) and 0.49 (0.24-0.98), compared with current smokers.

**Conclusions:**

Cumulative smoking exposure and duration of smoking cessation were significantly associated with PAD in middle aged and older Korean men.

## Background

Peripheral arterial disease (PAD), one of the major manifestations of generalized atherosclerotic disease, results from a narrowing of arteries in the lower extremities, as a result of progressive atherosclerosis [1]. Although PAD is a marker of coronary and cerebral atherosclerotic vascular disease, PAD is a commonly overlooked condition in primary care settings, because most patients are asymptomatic. However, people with PAD, even if asymptomatic, have an increased risk of future cardiovascular events and related mortality [2-4]. The ankle-brachial index (ABI), the ratio of systolic blood pressure in the ankle to that in the arm, is a simple, reproducible, and non-invasive test to diagnose PAD and has been used to diagnose and evaluate the severity of PAD in the lower extremities [5].

Cigarette smoking contributes to the constriction and damage of arteries and is a potent risk factor for PAD, by promoting endothelial dysfunction and by altering lipoprotein metabolism, coagulation, and platelet function [6]. A number of epidemiological studies have reported an association between PAD and both current and former smoking [7-14], though few studies have examined the cumulative effects of smoking, such as pack-years exposure, on PAD [15]. Moreover, because few studies have examined the association between smoking cessation and PAD [13], it remains undetermined whether the duration of smoking cessation is inversely associated with PAD in the general population.

The objective of our study was to investigate the association of cumulative cigarette smoking exposure with PAD prevalence among community-dwelling adults aged 50 years and older, and to determine whether the duration of smoking cessation was inversely associated with PAD.

## Methods

### Subjects

The study population consisted of community-dwelling men and women aged 50 years and older who participated in the 2007-2009 baseline surveys of the Dong-gu Study [16]. We used the national resident registration records to identify potential participants. In total, 26,323 eligible subjects aged 50 years and over who resided in one of five towns in the Dong-gu district of Gwangju Metropolitan City in South Korea were invited by telephone to participate. Total 6779 subjects were enrolled and the response rate for this study was 25.8% (21.8% for men and 29.1% for women). However, only men were included in the analysis because of the low prevalence of smoking in women (1.9% current smokers and 1.8% former smokers). Of the 2644 men, 100 subjects were excluded from the study because of incomplete medical histories, lifestyle characteristics, and anthropometric and biochemical measurements. An additional 21 subjects were excluded because of missing information on the ABI measurements. Six individuals with an ABI greater than 1.50 were further excluded from the analysis because this indicates poorly compressible leg arteries and the inability to gauge arterial perfusion accurately [17,18]. In total, 2517 subjects were included in the analyses. This study was conducted in accordance with the Declaration of Helsinki guidelines and the study protocol was approved by the institutional review board of Chonnam National University Hospital. Participants provided written informed consent.

### Definition of smoking

Participants were classified based on smoking habit information collected using a standardized questionnaire by well-trained research staff. Smoking was classified as never smokers (smoked <100 cigarettes in their lifetime and not currently smoking), former smokers (smoked ≥100 cigarettes in their lifetime and currently a non-smoker), and current smokers (smoked ≥100 cigarettes in their lifetime and currently a smoker) [10]. Cumulative smoking exposure in former and current smokers was determined in terms of pack-years by multiplying the number of years smoked with the average number of packs per day [15]. Based on pack-years of smoking, subjects were classified as never smokers (0.0 pack-years), light smokers (0.1-20.0 pack-years), moderate smokers (20.1-40.0 pack-years), and heavy smokers (> 40 pack-years). Based on information on years since quitting smoking, former smokers were classified as ≤3 years, 4-10 years, 11-20 years, and ≥21 years and compared with current smokers.

### ABI measurement and PAD definition

ABI, an indicator of PAD, was measured using an automated, non-invasive, waveform analysis device (VP-1000, Colin Co, Komaki, Japan). The subjects were examined in the supine position after relaxing in the supine position on a bed for at least 5 min. Cuffs were wrapped around both the arms and the ankles, and electrocardiogram electrodes were placed at the left sternal border. ABI was automatically calculated by dividing the posterior tibial systolic blood pressure by the brachial systolic pressure measured by the oscillometric method with cuffs adapted to the extremities. ABI was obtained for each leg separately, and the average was used as the mean ABI. PAD, or an abnormal ABI, was defined as an ABI of lower than 0.90 in either leg [10,19]. The absence of PAD was defined as an ABI ≥0.90 and ≤1.50 [17,20]. An ABI less than 0.90 shows 95% sensitivity and 99% specificity for diagnosing PAD, as confirmed by angiography [21].

### Other variables of interest

Information on the demographic characteristics, lifestyle, medical history, and medication use of each subject were assessed with a standardized questionnaire administered by trained staff. Alcohol consumption (g/day) was calculated from the average number of alcoholic beverages consumed. Physical exercise was categorized as none (0-1 time per week), irregular exercise (2-4 times per week), and regular exercise (5 or more times per week) [16]. Body mass index was calculated as weight in kilograms divided by height in meters squared. Blood pressure was measured after at least 5 min of rest in the sitting position using a mercury sphygmomanometer. The average of three consecutive readings of systolic and diastolic blood pressure taken at 1-min intervals was used in the analysis. After a 12-h overnight fast, venous blood samples were collected. Serum total cholesterol, high-density lipoprotein (HDL) cholesterol, triglycerides, uric acid, and fasting blood glucose levels were measured using enzymatic methods. Low-density lipoprotein (LDL) cholesterol was calculated by the Friedewald formula. All samples were analyzed using an automatic analyzer (Hitachi 7600, Hitachi Ltd., Tokyo, Japan).

### Statistical analysis

General characteristics of the study population, based on the presence or absence of PAD, were expressed as mean ± standard deviation or number (percentage), and were compared using independent *t*-tests for continuous variables and chi-squared tests for categorical variables. Analysis of covariance and multiple logistic regression were used to evaluate the association of smoking characteristics (e.g., smoking status, cumulative smoking exposure, and duration of smoking cessation) with mean ABI and PAD, respectively. Mean (standard error) or odds ratio (95% confidence interval) were calculated in unadjusted, age-adjusted, and multivariate-adjusted models adjusting for age, body mass index, systolic blood pressure, fasting blood glucose, total cholesterol, HDL cholesterol, uric acid, alcohol consumption, physical exercise, and use of medications for hypertension, diabetes, and hyperlipidemia. Multivariate-adjusted models of cumulative smoking exposure were further adjusted for current smoking (yes/no), and multivariate-adjusted models of duration of smoking cessation were additionally adjusted for pack-years of smoking. The trend in the association between smoking characteristics and PAD was determined after considering smoking categories as ordinal variables. All statistical analyses were performed using SPSS 15.0 and *P *values <0.05 were deemed to indicate statistical significance.

## Results

Among the 2517 men aged 50 years and older included in the analysis, 103 subjects had PAD (4.1%) and 2414 subjects were classified as normal (95.9%). General characteristics of the study population by the presence of PAD are presented in Table 1. Significant differences between those with and without PAD were observed in age, systolic blood pressure, uric acid, alcohol consumption, total years of smoking, pack-years of smoking, and the use of antihypertensive and antidiabetic medications. The mean ABI was 0.868 ± 0.117 in subjects with PAD and 1.147 ± 0.082 in subjects without PAD (*P *< 0.001).

**Table 1:** General characteristics of the study population with and without PAD (n = 2517)

	Without PAD (n = 2414)	With PAD (n = 103)	*P*-value^a^
Age, years	65.9 ± 7.9	70.4 ± 7.3	<0.001
Body mass index, kg/m²	23.9 ± 2.8	23.4 ± 3.0	0.118
Systolic blood pressure, mmHg	124.8 ± 16.3	129.6 ± 19.5	0.014
Diastolic blood pressure, mmHg	75.7 ± 10.5	74.8 ± 10.0	0.403
Antihypertensive medications, n (%)	813 (33.7)	53 (51.5)	<0.001
Fasting blood glucose, mg/dl	113.3 ± 27.2	116.8 ± 33.1	0.205
Antidiabetic medications, n (%)	362 (15.0)	34 (33.0)	<0.001
Total cholesterol, mg/dl	187.7 ± 37.7	189.9 ± 38.3	0.567
LDL cholesterol, mg/dl	109.7 ± 35.7	114.0 ± 37.1	0.231
HDL cholesterol, mg/dl	49.3 ± 12.0	47.1 ± 10.0	0.073
Triglycerides, mg/dl^b^	143.7 ± 116.5	144.1 ± 96.4	0.977
Lipid-lowering medications, n (%)	150 (6.2)	6 (5.8)	0.873
Uric acid, mg/dl	5.8 ± 1.4	6.4 ± 1.9	0.001
Alcohol consumption, gram/day	14.8 ± 23.7	10.1 ± 20.0	0.023
Regular exercise, n (%)	492 (20.5)	16 (15.7)	0.239
Age at starting smoking	22.4 ± 5.6	22.9 ± 6.5	0.427
Total years of smoking, years	31.9 ± 13.8	36.8 ± 14.9	0.001
Number of cigarettes per day	17.9 ± 12.0	19.1 ± 12.7	0.356
Pack-years of smoking	21.5 ± 22.2	31.3 ± 29.7	0.001
Mean ankle-brachial index	1.147 ± 0.082	0.868 ± 0.117	<0.001

Data are presented as means ± standard deviation or number (percentage).

PAD, peripheral arterial disease, was defined as an ankle-brachial index lesser than 0.90 in either leg; LDL, low-density lipoprotein; HDL, high-density lipoprotein.

^a^*P*-value was obtained by Student's t-test or by chi-square test.

^b^*P*-value was obtained after log-transformation.

Of all the subjects, 616 (24.5%) had never smoked, 1298 (51.6%) were former smokers, and 603 (24.0%) were current smokers. Mean ABI and OR for PAD by smoking status are shown in Table 2. Compared to never smoking, former and current smokers had significantly smaller mean ABI in the unadjusted, age-adjusted, and multivariate-adjusted models (*P *< 0.001 in all models). Moreover, current smokers had significantly smaller mean ABI than former smokers in all models. Former and current smokers had significantly higher risk for PAD than those who had never smoked. Compared with never smoking, the OR (95% CI) for PAD was 2.31 (1.20-4.42) for former smokers and 4.30 (2.13-8.66) for current smokers in the multivariate-adjusted model. Furthermore, the risk of PAD in current smokers was significantly higher than in former smokers in the multivariate-adjusted model (OR, 1.89; 95% CI, 1.19-3.01).

**Table 2:** Mean ankle-brachial index and odds ratio for peripheral arterial disease according to smoking status

	Smoking status		
	
	Never smokers (n = 616)	Former smokers (n = 1298)	Current smokers (n = 603)
Mean ankle-brachial index			
Unadjusted	1.156 (0.004)	1.134 (0.003)***	1.118 (0.004)***††
Age-adjusted	1.157 (0.004)	1.134 (0.003)***	1.116 (0.004)***†††
Multivariate-adjusted^a^	1.153 (0.004)	1.134 (0.003)***	1.120 (0.004)***††
Peripheral arterial disease^b^			
Prevalence, n (%)	12 (1.9)	55 (4.2)	36 (6.0)
Unadjusted OR	1.00 (reference)	2.23 (1.18-4.19)*	3.20 (1.65-6.20)**
Age-adjusted OR	1.00 (reference)	2.31 (1.22-4.35)*	4.29 (2.18-8.43)***
Multivariate-adjusted^a ^OR	1.00 (reference)	2.31 (1.20-4.42)*	4.30 (2.13-8.66)***
Unadjusted OR	-	1.00 (reference)	1.44 (0.93-2.21)
Age-adjusted OR	-	1.00 (reference)	1.87 (1.20-2.92)††
Multivariate-adjusted^a ^OR	-	1.00 (reference)	1.89 (1.19-3.01)††

Data are presented as mean (standard error) or odds ratio (95% confidence interval).

^a^Adjusted for age, body mass index, systolic blood pressure, fasting blood glucose, total cholesterol, HDL cholesterol, uric acid, alcohol consumption, physical exercise, and use of medications for hypertension, diabetes, and hyperlipidemia.

^b^Defined as an ankle-brachial index lesser than 0.90 in either leg.

*P < 0.05, **P < 0.01, ***P < 0.01; compared with never smokers.

†*P *< 0.05, ††*P *< 0.01, †††*P *< 0.01; compared with former smokers.

Table 3 shows the mean ABI and OR for PAD by cumulative smoking exposure. A significant decrease in mean ABI with pack-years of smoking was observed in all models (*P-*trend <0.001 in all models). Additionally, compared with never smoking, light, moderate, and heavy smokers had significantly lower ABI in all models. Significant increases in PAD risk with pack-years of smoking were observed in all models (*P-*trend <0.001, <0.001, and = 0.008, respectively). In the multivariate-adjusted model, compared with those who had never smoked, the OR for PAD was 2.15 (1.06-4.38), 2.24 (1.08-4.65), and 2.93 (1.41-6.09) in light, moderate, and heavy smokers, respectively.

**Table 3:** Mean ankle-brachial index and odds ratio for peripheral arterial disease according to cumulative smoking exposure

	Never smokers (n = 616)	Cumulative smoking exposure (pack-years of smoking)^c^			*P-*trend^d^
			
		Light smokers [0.1-20.0 pack-years] (n = 745)	Moderate smokers [20.1-40.0 pack-years] (n = 687)	Heavy smokers [> 40.0 pack-years] (n = 438)	
Mean ankle-brachial index					
Unadjusted	1.156 (0.004)	1.138 (0.004)**	1.125 (0.004)***	1.118 (0.005)***	<0.001
Age-adjusted	1.156 (0.004)	1.138 (0.004)**	1.124 (0.004)***	1.119 (0.005)***	<0.001
Multivariate-adjusted^a^	1.150 (0.004)	1.137 (0.004)*	1.128 (0.004)***	1.123 (0.005)***	<0.001
Peripheral arterial disease^b^					
Prevalence, n (%)	12 (1.9)	30 (4.0)	31 (4.5)	30 (6.8)	
Unadjusted OR	1.00 (reference)	2.11 (1.07-4.16)*	2.38 (1.21-4.67)*	3.70 (1.87-7.31)***	<0.001
Age-adjusted OR	1.00 (reference)	2.32 (1.17-4.59)*	2.85 (1.44-5.64)**	3.69 (1.86-7.31)***	<0.001
Multivariate-adjusted^a ^OR	1.00 (reference)	2.15 (1.06-4.38)*	2.24 (1.08-4.65)*	2.93 (1.41-6.09)**	0.008

Data are presented as mean (standard error) or odds ratio (95% confidence interval).

^a^Adjusted for age, body mass index, systolic blood pressure, fasting blood glucose, total cholesterol, HDL cholesterol, uric acid, alcohol consumption, physical exercise, use of medications for hypertension, diabetes, and hyperlipidemia, and current smoking (yes/no).

^b^Defined as an ankle-brachial index lesser than 0.90 in either leg.

^c^Thirty-one smokers were excluded due to lack of information about pack-years of smoking.

^d^*P*-trend estimated from analysis of covariance or logistic regression using the categories of cumulative smoking exposure as an ordinal variable.

*P < 0.05, **P < 0.01, ***P < 0.001; compared with never smokers.

Table 4 shows the mean ABI and OR for PAD by years since quitting smoking. A significant dose-response association was found between years since quitting smoking and mean ABI in all models (*P-*trend <0.001). Former smokers with 11-20 and ≥21 years of smoking cessation had significantly greater mean ABI than individuals who had never smoked in all models. Risk for PAD decreased with increasing years since quitting smoking in all models (*P-*trend = 0.002, <0.001, and <0.001, respectively). The risk of PAD in former smokers with 11-20 years and ≥21 years of smoking cessation was significantly lower than in current smokers in all models, and the OR was 0.41 (0.19-0.86) and 0.49 (0.24-0.98) in the multivariate-adjusted models, respectively.

**Table 4:** Mean ankle-brachial index and odds ratio for peripheral arterial disease according to duration of smoking cessation

	Current smokers (n = 603)	Duration of smoking cessation (years since quitting smoking)^c^				Never smokers (n = 616)	*P-*trend^d^
				
		≤ 3 years (n = 185)	4-10 years (n = 398)	11-20 years (n = 303)	≥ 21 years (n = 357)		
Mean ankle-brachial index							
Unadjusted	1.118 (0.004)	1.127 (0.007)	1.130 (0.005)	1.140 (0.006)**	1.138 (0.005)**	1.156 (0.004)***	<0.001
Age-adjusted	1.116 (0.004)	1.125 (0.007)	1.129 (0.005)*	1.141 (0.006)***	1.141 (0.005)***	1.157 (0.004)***	<0.001
Multivariate-adjusted^a^	1.123 (0.004)	1.132 (0.008)	1.134 (0.005)	1.139 (0.006)*	1.138 (0.005)*	1.147 (0.005)***	<0.001
Peripheral arterial disease^b^							
Prevalence, n (%)	36 (6.0)	6 (3.2)	17 (4.3)	10 (3.3)	16 (4.5)	12 (1.9)	
Unadjusted OR	1.00 (reference)	0.53 (0.22-1.27)	0.70 (0.39-1.27)	0.54 (0.26-1.10)	0.74 (0.40-1.35)	0.31 (0.16-0.61)**	0.002
Age-adjusted OR	1.00 (reference)	0.49 (0.20-1.19)	0.59 (0.32-1.08)	0.41 (0.20-0.84)*	0.47 (0.25-0.88)*	0.23 (0.12-0.45)**	<0.001
Multivariate-adjusted^a ^OR	1.00 (reference)	0.47 (0.19-1.17)	0.57 (0.30-1.06)	0.41 (0.19-0.86)*	0.49 (0.24-0.98)*	0.25 (0.11-0.55)**	<0.001

Data are presented as mean (standard error) or odds ratio (95% confidence interval).

^a^Adjusted for age, body mass index, systolic blood pressure, fasting blood glucose, total cholesterol, HDL cholesterol, uric acid, alcohol consumption, physical exercise, use of medications for hypertension, diabetes, and hyperlipidemia, and pack-year of smoking.

^b^Defined as an ankle-brachial index lesser than 0.90 in either leg.

^c^Fifty-five former smokers were excluded due to lack of information about years since quitting smoking.

^d^*P*-trend estimated from analysis of covariance or logistic regression using the categories of years since quitting smoking as an ordinal variable.

*P < 0.05, **P < 0.01, ***P < 0.001; compared with current smokers.

## Discussion

This cross-sectional study of community-dwelling Korean men demonstrated that cumulative smoking exposure was positively associated with PAD, defined as an ABI <0.90, and that duration of smoking cessation was inversely associated with PAD, independently of conventional cardiovascular risk factors. Although, as in previous studies, current smokers had a significantly higher risk for PAD than those who never or previously smoked, pack-years of smoking and years since quitting smoking were also identified as significant risk factors that should be taken into account to properly evaluate the effect of cigarette smoking on PAD.

The ABI is considered as a surrogate marker of generalized atherosclerosis because low ABI levels have been associated with elevated risk of future coronary heart disease [3,22], stroke [3,23,24], and a higher risk of all-cause and cardiovascular mortality [3,25,26]. A recent meta-analysis found that the predictive value of ABI for cardiovascular morbidity and mortality was similar to that of the traditional Framingham risk factors [27]. Because ABI is simple, non-invasive, reproducible, and cost-effective [28], ABI measurement is routinely performed to screen for asymptomatic PAD patients. ABI is a useful tool for the prediction of cardiovascular risk because, compared with carotid intima-media thickness and coronary artery calcium, it can be performed readily in community settings and in primary care physicians' offices [28].

Our results are consistent with previous findings that current smoking is associated with a 1.86 to 5.48-fold increase in risk of PAD, compared with never having smoked [7-14]. Some studies have reported that current smoking is associated with a 1.60 to 2.80-fold increase in the risk of PAD, compared with never or previously smoking [29,30]. Additionally, our findings are analogous to previous findings that former smoking is associated with a 1.02 to 1.94-fold increased risk of PAD [7,9-11,14]; however, generally no significant difference between former and never smoking has been found.

Although various epidemiological studies have reported an association between smoking status and PAD, the majority of studies have examined PAD risk by smoking status, finding that current smoking increased PAD risk or smoking cessation decreased PAD risk. However, the simple smoking status classification of never, former, and current smoking has been questioned on the assumption that lifetime heavy smokers who have recently stopped smoking are categorized as former smokers, whereas smokers who started smoking only a few months ago are categorized as current smokers. Thus, cumulative smoking exposure, such as pack-years, should be taken into account to properly evaluate the association between cigarette smoking and PAD. Because few studies have investigated the association between cumulative smoking exposure and PAD or between the duration of smoking cessation and PAD, the dose-response relationship between smoking habits (e.g., pack-years of smoking and years since quitting smoking) and PAD is uncertain. The Edinburgh Artery Study of 1592 adults reported that the adjusted relative risk of PAD, compared with never smoking, was 1.70 (95% CI, 0.72-3.99) for moderate smokers (≤25 pack-years) and 2.72 (95% CI, 1.13-6.53) for heavy smokers (> 25 pack-years) [15]. Recently, a cross-sectional study of 1215 Japanese men found that mean ABI correlated inversely and linearly with pack-years of smoking and the OR (95% CI) of PAD, compared with never smoking, was 2.8 (0.8-10.2), 2.8 (0.8-10.0), and 4.2 (1.2-14.6) for <26, 26-45, and ≥45 pack-years, respectively, suggesting a linear trend [13]. In agreement with previous studies [13,15], we observed a significant increasing trend between pack-years of smoking and PAD, confirming that the effect of cumulative smoking exposure on PAD was a dose-response relationship. Cui et al. [13] found that men who had quit smoking 20 or more years ago had higher mean ABI and lower prevalence of PAD than current smokers. In our study, the risk of PAD was significantly lower with smoking cessation of over 11 years than in current smokers, whereas ≤10 years of smoking cessation was not significantly associated with PAD risk, suggesting that long-term smoking cessation is needed to diminish the effects of smoking on PAD.

### Study limitations and strengths

There are several limitations to the present study. First, the cross-sectional nature limits conclusions about the direction or causality of associations observed in our study. Additional prospective studies with incident PAD are needed to confirm our findings. Second, the possible measurement errors in smoking characteristics due to the information being self-reported, without measuring biological markers, such as serum cotinine, might have attenuated the relationship between cigarette smoking and PAD. Third, unmeasured confounding variables could have affected the association of cigarette smoking and smoking cessation with PAD. Fourth, because information on environmental tobacco smoke exposure was not collected, the effect of passive smoking on PAD cannot be accessed.

Nevertheless, this study has several strengths. First, the main strength is that few studies have investigated the association between smoking characteristics (including pack-years of smoking and years since quitting smoking) and PAD risk. Our more detailed analysis, compared with previous studies, allowed us to find a significant association between smoking habits and PAD. Second, ABI was measured on both the left and right sides, and the smallest ABI was used in defining PAD. Third, a large number of community-dwelling older men participated in this study. Because subclinical atherosclerosis progresses with age, the present study population (aged 50 years and older) may help detect an association between smoking habits and PAD.

## Conclusions

In summary, a significant dose-response association between cumulative smoking exposure and PAD prevalence was found in a community-dwelling sample of Korean men. Additionally, there was a significant inverse association between duration of smoking cessation and presence of PAD. From a public health perspective, considering the high prevalence of current smoking in Korean men, more aggressive tobacco control efforts could reduce the number of people who develop PAD.

## Competing interests

The authors declare that they have no competing interests.

## Authors' contributions

YHL carried out physical examinations, analyzed the data, and drafted the manuscript. MHS and SSK coordinated the data collection and also performed physical examinations. HRA, WJY, SYR, BHK, HSN, SKJ, and KSP conducted physical measurements and collected data. JSC and JAR participated in the design of the study, the data collection, and reviewed the manuscript. All authors read and approved the final manuscript to be published.

## Pre-publication history

The pre-publication history for this paper can be accessed here:

http://www.biomedcentral.com/1471-2458/11/94/prepub
